# Mannosylated Mucin-Type Immunoglobulin Fusion Proteins Enhance Antigen-Specific Antibody and T Lymphocyte Responses

**DOI:** 10.1371/journal.pone.0046959

**Published:** 2012-10-12

**Authors:** Gustaf Ahlén, Lena Strindelius, Tomas Johansson, Anki Nilsson, Nathalie Chatzissavidou, Magnus Sjöblom, Ulrika Rova, Jan Holgersson

**Affiliations:** 1 Recopharma AB, Stockholm, Sweden; 2 Biochemical and Chemical Process Engineering, Luleå University of Technology, Luleå, Sweden; 3 Clinical Chemistry and Transfusion Medicine, Sahlgrenska Academy, Gothenburg, Sweden; Federal University of São Paulo, Brazil

## Abstract

Targeting antigens to antigen-presenting cells (APC) improve their immunogenicity and capacity to induce Th1 responses and cytotoxic T lymphocytes (CTL). We have generated a mucin-type immunoglobulin fusion protein (PSGL-1/mIgG_2b_), which upon expression in the yeast *Pichia pastoris* became multivalently substituted with O-linked oligomannose structures and bound the macrophage mannose receptor (MMR) and dendritic cell-specific intercellular adhesion molecule-3 grabbing non-integrin (DC-SIGN) with high affinity *in vitro*. Here, its effects on the humoral and cellular anti-ovalbumin (OVA) responses in C57BL/6 mice are presented.

OVA antibody class and subclass responses were determined by ELISA, the generation of anti-OVA CTLs was assessed in ^51^Cr release assays using *in vitro*-stimulated immune spleen cells from the different groups of mice as effector cells and OVA peptide-fed RMA-S cells as targets, and evaluation of the type of Th cell response was done by IFN-γ, IL-2, IL-4 and IL-5 ELISpot assays.

Immunizations with the OVA − mannosylated PSGL-1/mIgG_2b_ conjugate, especially when combined with the AbISCO®-100 adjuvant, lead to faster, stronger and broader (with regard to IgG subclass) OVA IgG responses, a stronger OVA-specific CTL response and stronger Th1 and Th2 responses than if OVA was used alone or together with AbISCO®-100. Also non-covalent mixing of mannosylated PSGL-1/mIgG_2b_, OVA and AbISCO®-100 lead to relatively stronger humoral and cellular responses. The O-glycan oligomannoses were necessary because PSGL-1/mIgG_2b_ with mono- and disialyl core 1 structures did not have this effect.

Mannosylated mucin-type fusion proteins can be used as versatile APC-targeting molecules for vaccines and as such enhance both humoral and cellular immune responses.

## Introduction

Targeting antigens to endocytic receptors on professional antigen-presenting cells represents an attractive strategy to enhance the efficacy of vaccines. Mannosylated antigens have been demonstrated to enhance MHC class I- and MHC class II-restricted antigen presentation, increase T-cell proliferation, and promote T cell effector responses [1], [2], [3] through mannose-mediated binding to endocytic receptors on dendritic cells and macrophages [4]. These receptors belong to the family of calcium-dependent C-type lectin receptors and include the mannose receptor (MR) and dendritic cell-specific intracellular adhesion molecule-3 grabbing non-integrin (DC-SIGN). They are particularly designed to sample antigen (self and non-self), much like pattern recognition receptors, and to integrate innate and adaptive immune responses [5], [6], [7]. DC-SIGN is mainly expressed on immature and mature dendritic cells (DCs) with crucial functions in DC trafficking and T-cell interactions as well as pathogen recognition [8]. MR is expressed primarily by tissue macrophages, and lymphatic and hepatic endothelia in humans and mice [9], [10]. MR is also expressed by subsets of dendritic cells (DC), primarily interstitial DCs, as well as on cultured DCs from human monocytes [11], [12].

Several studies have shown that polymeric mannose (mannan) improve antigen presentation and that oxidized and reduced mannan can induce antigen-specific Th1/CTL and Th2/humoral responses, respectively [13], [14], [15], [16]. Tumor immunity was induced with the generation of both antigen-specific humoral and cellular immunity in hMR transgenic mice by antigenic targeting to MR [17]. Yeast-derived recombinant ovalbumin (OVA) carrying branched N- and O-linked mannoses were shown to be far more immunogenic than non-mannosylated OVA [18]. Enhancement of antigen uptake was achieved by extensive O-mannosylation of proteins, and deglycosylation strongly inhibited T cell responses [19]. Furthermore, MR-mediated endocytosis of OVA has been shown to be essential for cross-presentation to CD8+ T cells [20].

In contrast to protein-protein interactions, individual protein-carbohydrate interactions are characterized by a low affinity binding with Kd values many times in the mM range [21], [22], [23]. Instead the overall binding strength in such interactions is to a large extent accomplished by multivalency, *i.e.* several carbohydrate determinants on for example a cell binding several copies of a carbohydrate-specific receptor or domain on another cell. Multivalency can increase the affinity of a particular protein-carbohydrate interaction several thousand-fold resulting in nano- and picomolar Kd values [24], [25]. This is exemplified by the asialoglycoprotein receptor, which recognizes serum glycoproteins with increasing affinity as the number of exposed terminal galactoses increases with age [26]. In addition to multivalency, the molecular fit between the carbohydrate and its receptor can add to the affinity. In this respect the fact that both the inner core saccharide chain as well as the protein backbone may influence the presentation of the carbohydrate determinant is of particular importance [27], [28], [29].

P-selectin glycoprotein ligand-1/mouse IgG_2b_ (PSGL-1/mIgG_2b_) is a mucin-like immunoglobulin fusion glycoprotein with 106 potential sites for O-linked glycosylation and six potential sites for N-linked glycosylation in its dimeric form [30]. With the aim of interfering with or promoting protein-carbohydrate interactions of biomedical importance, we have used this fusion protein as a scaffold for multivalent presentation of various tailored carbohydrate determinants of diagnostic or therapeutic significance [30]. By displaying multiple oligomannose chains in various combinations for MR, DC-SIGN and mannan binding lectin (MBL), we have shown that recombinant PSGL-1/mIgG_2b_ produced in the yeast *Pichia pastoris* can target these receptors with high affinity by engaging multiple carbohydrate recognition domains of MR and MBL or multiple/oligomerized DC-SIGN receptors [31].

We hypothesize that a mucin-type fusion protein carrying multiple O-linked oligomannose structures has the potential of working as a universal antigen presenting cell (APC)-targeting molecule for a broad repertoire of protein antigens in different vaccine compositions. As such, it may amplify both humoral and cellular immune responses and may be used together with different antigens for which already established manufacturing bioprocesses can be maintained. Here, we present data on the OVA-specific immune responses in mice immunized with OVA, OVA-mannosylated PSGL-1/mIgG_2b_ conjugates or mixtures, with or without an additional adjuvant in the form of Imject®Alum or AbISCO®-100.

## Materials and Methods

### Mice

Inbred C57Bl/6J (H-2^b^) mice were bred and housed at Karolinska Institutet, Division of Comparative Medicine, Clinical Research Center, Karolinska University Hospital, Huddinge. The animals were caged at five to ten mice per cage and fed a commercial diet with free access to food and water. All animals were six to eight weeks of age at the start of the experiment.

### Ethics Statement

All mice were bred and maintained according to the regulations of the Ethical Committee for Animal Research at Karolinska Institutet. All animal experiments were approved by the regional committee (Stockholms södra djuretiska nämnd) on animal ethics, S-184-06 and S-132-09.

### Proteins, peptides and adjuvants

For ELISpot and proliferation assays different proteins and peptides at varying concentrations were used: the OVA-SIINFEKL (MHC Class I) CTL peptide (Innovagen, Lund, Sweden, OVA 257-264, SP-O257-5) was used at a concentration of 1 µg/mL – 0.0001 µg/mL, the FILKSINE control (MHC Class I) CTL peptide (Innovagen, SP-CS) at 1 µg/mL, the Th-OVA (MHC Class II) Th peptide (Innovagen, OVA 323-339, SP-O323A-5) at 10 µg/mL – 0.01 µg/mL, the OVA protein grade VII (Sigma Aldrich, St Louis, MO, USA, A7641) was used at a concentration of 625 µg/mL – 5 µg/mL, BSA (Sigma Aldrich, A8806) at 25 µg/mL and Concanavalin A (Sigma Aldrich, L7647) at 5 and 1 µg/mL.

The LC-SPDP linker (Pierce) and Imject Alum were from Thermo Fischer Scientific (Waltham, MA, USA) and AbISCO®-100 was from Isconova AB (Uppsala, Sweden).

### Production of PSGL-1/mIgG_2b_

Mannosylated PSGL-1/mIgG_2b_ (PPM) was produced in *P. pastoris*, purified, quantified and characterized by Western blotting and mass spectrometry as described [31].

PSGL-1/mIgG_2b_ with mono and disialylated core 1 structure (CP) was produced in a stable CHO (Chinese hamster ovary) cell line given the name, C-P55.2. The cells were cultured in serum-free ProCHO4 medium (Lonza, Basel, Switzerland) in repeated batch mode in a 20 L Wave bioreactor (Wave System 20/50 EH, GE Health Care, Uppsala, Sweden). The bioreactor was inoculated at 0.8×10^6^ viable cells/mL in a volume of 5.2 L. At regular intervals, fresh cultivation medium with 2 mM glutamine was added as a bolus until the final volume in the bioreactor reached 10.3 L. The culture was harvested when the final cell density was 4.6×10^6^ total cells/mL and the viability dropped to 88%. The glucose, glutamine and pH levels were monitored daily and adjusted to optimal levels. Total cultivation time was 11 days.

Cell culture supernatants were clarified using a 540 cm^2^ Millistak+ POD C0HC filter (Millipore, Billerica, MA, USA) connected to the Quattroflow pump on the Cogent M TFF system (Millipore). The clarified supernatant was subsequently concentrated 22× using a 0.11 m^2^ Pellicon 3 ultrafilter (Millipore) on the Cogent M TFF system, and then further diafiltered against six volumes of PBS. Finally, 1 mL/L of protease inhibitor cocktail (Sigma Aldrich, P8215) and 0.02% NaN_3_ (Sigma Aldrich, 71289) were added to the product solution, which was stored at 4°C until purification.

All chromatographic procedures were carried out on an ÄKTAExplorer 100 (GE Healthcare) controlled by the Unicorn software (v. 5.11). The clarified supernatants were sterile filtered with a 0.22 µm polyethersulfone (PES) filter (TPP, Trasadingen, Switzerland) before loading onto a MabSelect SuRe column (GE Healthcare) pre-equilibrated with PBS. The column was washed with 10 column volumes (CV) of PBS, and elution of recombinant fusion protein was achieved using 5 CV of 0.1 M sodium citrate, pH 3.0. After elution, selected fractions were pooled, neutralized with 250 µL per mL of 1 M Tris-HCl, pH 9.0 and then dialyzed extensively (12–14 kDa cut-off) against MilliQ water at 4°C. After dialysis, the samples were frozen, lyophilized and stored at −80°C before further purification.

Lyophilized samples were dissolved at a concentration of approximately 5 mg/mL in gel filtration buffer (0.1 M sodium phosphate pH 7.2, 0.5 M sodium chloride). Gel filtration of PSGL-1/mIgG_2b_ was carried out on a pre-equilibrated HiPrep 26/60 Sephacryl S-300 HR column (GE Healthcare, 17-1196-01). Typically, 5 mL of sample was applied to the gel filtration column and eluted with a flow rate of 1 mL/minute. Eluted fractions were kept at 4°C until pooling was done on the basis of Western blot analysis. Pooled fractions were dialyzed as above, frozen, lyophilized and stored at −80°C.

### Conjugation of PSGL-1/mIgG_2b_ to ovalbumin

A 5 mg/mL (113 µM) solution of OVA was prepared in conjugation buffer (0.1 M sodium phosphate pH 7.2, 0.15 M sodium chloride, 1 mM EDTA). A 20 mM solution of the LC-SPDP linker was prepared in MilliQ water immediately prior to activation of OVA. For activation of OVA, 300 µL of the linker solution was added to 2.1 mL of OVA solution at a 25× molar excess of linker. The mixture was allowed to react under rotation for 30 minutes at room temperature, yielding activated OVA (OVA*). The solution was applied to a PD-10 desalting column (GE Healthcare, 17-0851-01) and OVA* eluted in 3.5 mL conjugation buffer thereby removing free linker. The eluted OVA* had a concentration of 3 mg/mL (68 µM) as determined with the BCA method (Pierce, 23225) using BSA (Sigma Aldrich) as standard.

A 10 mg/mL (67 µM) solution of the PSGL-1/mIgG_2b_ fusion protein was prepared in conjugation buffer. To obtain the fusion protein in a form suitable for conjugation with OVA*, the fusion protein solution was reduced with 100× molar excess of 0.5 M dithiothreitol (DTT, Fluka, 43815) solution. The reduction was carried out in a heat block at 37°C for 15 minutes. The DTT was removed from the fusion protein by passing the reaction solution twice through a PD MiniTrap G25 column (GE Healthcare).

To assess the amount of linker present on OVA*, a pyridine-2-thione assay was performed. 50 µL OVA* was mixed with 950 µL PBS in a plastic cuvette and the absorbance at 343 nm was measured. 2 µL 0.5 M DTT was added to the cuvette to cleave the chromophore from the LC-SPDP linker. After 15 minutes at room temperature the absorbance at 343 nm was measured again. The molecular substitution ratio (MSR) was calculated as follows:
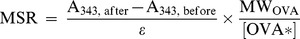
The reactions were repeated once. The activation of OVA for conjugation with PPM (study A), PPM (study B) and CP afforded an MSR of 6.0, 6.2 and 5.6, respectively. This indicates that there were on average 6.0, 6.2 and 5.6 linkers, respectively, on each OVA molecule for the PPM (study A and B) and CP conjugations.

To estimate the number of accessible thiol groups on the reduced fusion protein, Ellman's assay was performed. 2 µL reduced fusion protein, 998 µL Ellman's buffer (0.1 M sodium phosphate pH 8.0, 1 mM EDTA) and 50 µL 10 mM Ellman's reagent (5,5′-Dithio-*bis*-[2-nitrobenzoic acid], Pierce, 22582) was mixed in a cuvette. A blank was prepared in the same way, replacing the fusion protein with buffer. After 15 minutes at room temperature the absorbance at 412 nm was measured. Duplicate samples were analyzed. The concentration of accessible thiols was calculated as follows:
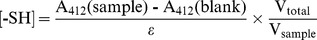
Dividing [-SH] with the concentration of reduced, monomeric fusion protein, a calculated number of 10.4 (PPM study A), 7.8 (PPM study B) and 5.9 (CP) thiol groups per monomer of fusion protein was obtained (8 cysteines in each monomer).

For conjugation, 2.816 mL OVA* and 1.0 mL reduced PPM (study A), 3.15 mL OVA* and 0.8 mL reduced PPM (study B), and 2.412 mL OVA* and 1 ml reduced CP, respectively, were mixed and split into two parallel reactions. The reaction was carried out at room temperature over night under rotation. See Table 1 for the molar quantities of the respective species for the different conjugations.

**Table 1:** Molar ratios of OVA to fusion protein and coupling yield for the conjugation.

	Study A	Study B	
	PPM	PPM	CP
Amount fusion protein reduced	48 nmol	67 nmol	41 nmol
Amount fusion protein in conjugation	48 nmol	54 nmol	41 nmol
Amount OVA activated	237 nmol	237 nmol	212 nmol
Amount LC-SPDP linker	6 µmol	6 µmol	5.4 µmol
Linker∶OVA ratio	25∶1	25∶1	25∶1
Amount OVA* in conjugation	192 nmol	214 nmol	164 nmol
OVA*∶fusion protein molar ratio	4∶1	4∶1	4∶1
Yields			
**OVA**-fusion protein	70%	22%	17%
OVA-**fusion protein**	Not analysed	76%	46%
OVA∶fusion protein molar ratio	-	1.1∶1	1.5∶1

After the conjugation reaction, the sample was centrifuged at 4,250× g and applied to a 26/60 HiPrep Sephacryl S300 gel filtration column (GE Healthcare) pre-equilibrated with 0.1 M sodium phosphate pH 7.2 with 0.5 M sodium chloride. Eluted fractions were kept at 4°C until pooling was done on the basis of Western blot analysis. Pooled fractions were then dialyzed as above, frozen, lyophilized and stored at −80°C.

### Quantification of OVA and fusion proteins

Quantification of conjugated OVA used for immunizations in study A was done by anti-OVA Western blot analysis using OVA of known concentration as standard. The OVA standard was determined with the BCA method using BSA as standard. The concentration of OVA in the stock solution was 2.0 mg/mL. A dilution series of DTT-reduced samples was heat-inactivated for 10 minutes at 70°C prior to separation on a 4–12% Novex Bis-Tris gel (MES buffer, 200 V for 45 minutes, Invitrogen). Two identical SDS-PAGE gels were run, blotted and analyzed as described below. Blotting was performed in an Invitrogen iBlot device for 10 minutes using an iBlot Transfer Stack (Invitrogen, San Diego, CA, USA, SKU# IB1001EU) with a nitrocellulose membrane (Invitrogen, SKU# IB3010-01). After washing of the membrane in PBS-Tween (PBS-T; 2×5 minutes), it was incubated in blocking solution (3% BSA in PBS-T) for one hour at room temperature (RT). After additional washes (3×5 minutes), the membrane was incubated over night at +4°C with an anti-OVA antibody (Sigma Aldrich, A6075) diluted 1∶20,000 in 3% BSA/PBS-T. The membrane was after washing (3×5 minutes in PBS-T) incubated for one hour at RT with a horseradish peroxidase (HRP)-conjugated secondary goat anti-mIgG Fab antibody (Sigma Aldrich, A2304) diluted 1∶25,000 in 3% BSA/PBS-T. The membranes were washed three times before they were incubated with chemo-luminescent HRP substrate for 5 minutes (Immobilon Western WBKLS0050; Millipore). The membranes were then exposed to Amersham Hyperfilm ECL for 30 seconds to 5 minutes for visualization of anti-OVA staining. The films were scanned using a Fluor-S MAX MultiImager (BioRad, Hercules, CA, USA) and the concentration of OVA released from the conjugate was quantified using the Quantity One v. 4.6.5 software (BioRad). The anti-OVA staining of the known OVA samples and the dilutions of the reduced conjugate were integrated and a standard curve was created. By fitting the integrated volumes of the anti-OVA staining of the reduced conjugate samples to the standard curve, the concentration of OVA in the conjugate estimated from the two gels was 2.4 and 2.5 mg/mL, respectively. With a volume of 2.5 mL this amounts to 6.0 mg of OVA conjugated to the PSGL-1/mIgG_2b_ fusion protein. The final amount of conjugated PSGL-1/mIgG_2b_ fusion protein was assumed to be equal to the amount applied in the conjugation reaction. The amounts in the conjugation and coupling yield for OVA for study A are shown in Table 1.

In study B, quantification of conjugated OVA, PPM and CP (in conjugates OVA−PPM and OVA−CP) used for immunizations was done by SDS-PAGE and SilverQuest analysis (Invitrogen, LC6070) using OVA, PPM and CP of known concentration as standard. The OVA standard was determined with the BCA method using BSA as standard. The concentration of OVA in the stock solution was 2.3 mg/mL. Concentrations of stock solutions of PPM and CP were determined by ELISA to 4 mg/mL and 0.33 mg/mL, respectively. A dilution series of DTT-reduced samples was heat inactivated for 10 minutes at 70°C prior to separation on a 4–12% Novex Bis-Tris gel (MES buffer, 200 V for 45 minutes). Two SDS-PAGE gels were run, stained and analysed as described below. SilverQuest staining was performed according to the manufacturer's instructions. Briefly, the gels were rinsed in ultrapure water and fixed in 40% ethanol and 10% acetic acid in Ultrapure water, for 20 minutes with gentle rotation. The gels were washed in 30% ethanol for 10 minutes. The ethanol was decanted and sensitizing solution added to the gels and incubated for 10 minutes. The sensitizing solution was decanted, the gels washed in 30% ethanol for 10 minutes, and further washed in ultrapure water for 10 minutes. The gels were incubated in staining solution for 25 minutes, after which it was decanted and the gels washed with ultrapure water for 30 seconds. The gels were incubated in developing solution for 8 minutes both for the fusion protein and the OVA gels. Stop solution was then immediately added to the gels that were agitated for 10 minutes. The stop/developing solution was decanted and the gels washed in ultrapure water for 10 minutes. The gels were scanned using the Fluor-S MAX MultiImager (BioRad) and the concentration of OVA, PPM and CP released from the conjugate was quantified by fitting the integrated volumes of the OVA/PPM/CP staining of the reduced conjugate samples to the standard curve. For OVA the concentrations were 3.8 mg/mL and 3.4 mg/mL for the OVA−PPM and OVA−CP conjugates used in study B, respectively. PPM in OVA−PPM was estimated to 11.3 mg/mL and CP in OVA−CP was estimated to 7.9 mg/mL. The amounts and coupling yields for the conjugations as well as the molar ratios of OVA to fusion protein are shown in Table 1. To measure the endotoxin level in the vaccine preparations, Limulus amoebocyte lysate tests were performed with the gel-clot method (Apotekens Produktion och Laboratorier, Stockholm, Sweden).
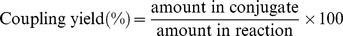


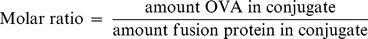



### Study design

Two separate studies were performed here described as study A and B. However, each experimental group has been repeated one to three times. In study A, 8 mice/group were immunized subcutaneously (s.c.) in the base of the tail using a dose of 50 µg OVA, either free or conjugated 1∶1 with mannosylated PSGL-1/mIgG_2b_ (PPM) produced in *Pichia pastoris*.

**Figure 1:**
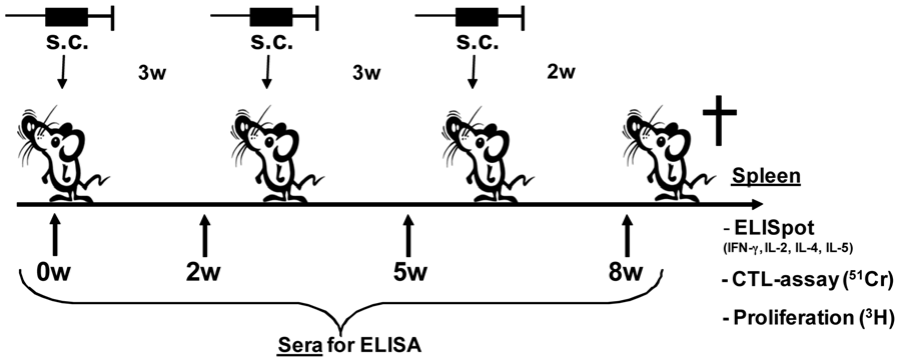
Experimental design. Schematic picture of the experimental design and the different read-outs used in study A and B.

The antigen and antigen conjugate were either given alone or in combination with AbISCO®-100 or Alum (Imject®). The mice were immunized three times with three-week intervals (Fig. 1). The day before the first immunization, mice under Isofluran (IsoFlo® vet, Orion Pharma) anesthesia were bled by retro-orbital bleeding. All immunizations were done under Hypnorm® (VetaPharma) anesthesia (20–30 µL intraperitonelly) by s.c. injections at the base of the tail vein. Mice were also bled two weeks after each immunization (w.2, w.5 and w.8). Boost immunizations were done at week three and five. Two weeks after the final immunization all mice were sacrificed and the spleen was recovered for cell isolation and further *in vitro* analyses of cellular immune responses (cell proliferation, cytokine ELISpot and CTL assays). The following groups of mice were included in study A: 1) 50 µg OVA in PBS (GIBCO, 10010-015); 2) 50 µg/140 µg OVA−PPM in PBS; 3) 50 µg OVA+12 µg AbISCO®-100 in PBS; 4) 50 µg/140 µg OVA−PPM+12 µg AbISCO®-100 in PBS; 5) 50 µg OVA in a 1∶1 mixture of PBS∶Imject® Alum; 6) 50 µg/140 µg OVA−PPM in a 1∶1 mixture of PBS∶Imject® Alum; and 7) 140 µg PPM in PBS. All material used for the immunizations had an endotoxin level below 1 EU/dose.

In study B, 8–10 mice/group were immunized as described above using a dose of 35 µg OVA either free or conjugated 1∶1 with mannosylated PSGL-1/mIgG_2b_ (PPM) produced in *P. pastoris* or with PSGL-1/mIgG_2b_ carrying mono and disialylated core 1 structures (CP) produced in CHO cells. The antigen and antigen conjugate were either given alone or in combination with AbISCO®-100. The same immunization scheme as described above was used (Fig. 1). The following groups of mice were included in study B: 1) 35 µg/89 µg OVA−PPM; 2) 35 µg/89 µg OVA−CP; 3) 35 µg OVA+12 µg AbISCO®-100; 4) 35 µg OVA+89 µg PPM+12 µg AbISCO®-100; 5) 89 µg PPM+12 µg AbISCO®-100; and 6) PBS (GIBCO, 10010-015). All groups had an endotoxin level below 1 EU/dose except the groups immunized with OVA−PPM that had 3.8–7.6 EU/dose and OVA−CP had 4.8–9.6 EU/dose.

### Detection of total IgG and IgG subclasses IgG1, IgG2a, IgG2b, IgG3 specific for OVA

Mouse sera analyzed with regard to antibody levels and isotypes were collected before the first immunization (w0) and then 2 weeks after each immunization (w.2, w.5 and w.8) by retro-orbital bleeding of isofluorane-anesthetized mice. To free the sera of cells, the samples were centrifuged twice at 6,000× g. OVA-specific mouse immunoglobulins were quantified by ELISA. ELISA plates (Corning, Lowell, MA, USA, 3590) were coated using a 10 µg/mL OVA (Sigma Aldrich, A7641) solution, which was incubated in the plates o/n at +4°C. After every incubation step the plates were washed 4 times with 400 µL of wash solution (9 g NaCl/L H_2_O+0.05% Tween). Plates were blocked with 1% BSA in PBS for 1 hour at 37°C. Serial dilutions of sera in 1% BSA/PBS were analyzed in duplicates or triplicates and incubated for 1 hour at 37°C. Anti-mouse IgG (Southern Biotech, Birmingham, AL, USA, 1030-05), IgG1 (Southern Biotech, 1070-05), IgG2a (Southern Biotech, 1080-05), IgG2b (Southern Biotech, 1090-05) or IgG3 (Southern Biotech, 1100-05)-HRP conjugates diluted 1∶2,000–8,000 and incubated for 1 hour at 37°C was used for detection of the different IgG subclasses. The TMB (tetramethylbenzidine, Sigma Aldrich, T3405) substrate (1 tablet) was dissolved in 10 mL phosphate citrate buffer, pH 5.0 containing 3 µL 30% H_2_O_2_ per 10 mL buffer and was used for detection of HRP conjugates. The reaction was stopped after 3–5 minutes with 2 M H_2_SO_4_. Optical density (OD) was measured in a TECAN Sunrise spectrophotometer (TECAN, Männedorf, Switzerland) at 450 nm within 2 hours after addition of H_2_SO_4_. An antibody titer was considered positive if the OD value was three times that of the animal serum collected prior to the first immunization. A hyperimmune serum of known titer was used as positive control. Pooled serum from non-immunized wt C57Bl/6J mice was used as negative control.

### Detection of mouse IgG

ELISA plates (Corning, Lowell, MA, USA, 3590) were coated using a 2 µg/mL PPM, CP or mIgG Fc fragment (Jackson ImmunoResearch, 015-000-008) o/n at +4°C. After every incubation step the plates were washed 4 times with 400 µL of wash solution (9 g NaCl/L H_2_O+0.05% Tween). Plates were blocked with 1% BSA in PBS for 1 hour at RT. Serial dilutions of pooled sera in 1% BSA/PBS were analyzed in duplicates and incubated for 2 hour at RT. An anti-mouse IgG Fab specific HRP antibody (Sigma, A2304) diluted 1∶5,000 and incubated for 2 hours at RT was used for detection. HRP conjugate was detected exactly as described in section 2.7. An antibody titer was considered positive if the OD value was three times that of the animal serum collected from the control group of mice only receiving PBS at each immunization.

### Detection of OVA-specific CTL responses

Spleens from OVA immunized C57Bl/6J mice were collected two weeks after the final immunization and single cell suspensions were prepared in RPMI-1640 medium containing 100 U/mL penicillin and 100 µg/mL streptomycin (GIBCO, Invitrogen, 15140). Red blood cells were removed using Red blood cell lysing buffer (Sigma Aldrich, R7757). Immune spleen cells (25×10^6^) were stimulated *in vitro* by co-cultivation in 25 mL-flasks containing 12 mL complete RPMI-1640 medium with 10% FBS (Sigma Aldrich, F7524), 100 U/mL penicillin and 100 µg/mL streptomycin (GIBCO, 15140), 10 mM HEPES (GIBCO, 15630), 2 mM L-glutamine (GIBCO, 25030), 1 mM nonessential amino acids (GIBCO, 1114035), 1 mM Sodium puruvate (GIBCO, 11360039) and 50 µM 2-mercaptoethanol (GIBCO, 31350) for 5 days with an equal number of irradiated (2,000 rad) syngeneic splenocytes and the MHC class I-specific SIINFEKL peptide at a concentration of 0.5 mM. Effector cells were harvested at day 5 and a 4 hour ^51^Cr release assay was performed in V-bottomed 96-well plates. RMA-S target cells (1×10^6^) were incubated with the SIINFEKL, or as negative control the FILKSINE, peptide at a concentration of 50 µM for 90 minutes at 37°C in a 5% CO_2_ atmosphere. The cells were carefully mixed every 15 minutes. Peptide-loaded target cells were incubated for 1 hour at 37°C with 30 µL ^51^Cr (5 mCi/mL, PerkinElmer, NEZ030005MC) and washed 3 times in PBS before use. Target cells were added to the plates in a total volume of 200 µL at effector∶target (E∶T) ratios of 80∶1, 40∶1 and 8∶1. The cytotoxic activity was determined after a 4 hour incubation at 37°C in a 5% CO_2_ atmosphere. Twenty-five µL supernatant were harvested and transferred to 200 µL OptiPhase super mix (PerkinElmer, Waltham, MA, USA; 1200-439) in a 96-well Isoplate (Wallac/PerkinElmer, 1450-514), and the radioactivity was measured in a γ-counter TRILUX 1450 MicroBeta counter (Wallac, PerkinElmer). Results were determined using the formula: percent specific lysis = (experimental release – spontaneous release)/(maximum release – spontaneous release). Experimental release is the mean count/minute released by target cells in the presence of effector cells. Maximum release was calculated from lysed incubated target cells. Spontaneous release was calculated from spontaneous release from incubated target cells. All samples were run in triplicates.

### Detection of OVA-specific CTLs and Th-cells producing IFN-γ, IL-2, IL-4 and IL-5

Spleen cells from four to five individual mice in each group were pooled and immediately tested for the presence of OVA-specific T cells. Spleen cells from the other four to five individuals in each group were used to repeat the experiment with consistent results. The ability of OVA-specific Th and CTLs to produce IFN-γ, IL-2, IL-4 and IL-5 after exposure to different peptides SIINFEKL (CTL, OVA_257–264_; Innovagen), FILKSINE (CTL, irrelevant peptide; Innovagen), and ISQAVHAAHAEINEAGR (Th, OVA_323–339_; Innovagen), proteins (OVA grade VII and BSA, Sigma Aldrich), Concanavalin-A (Sigma Aldrich) and media was assessed. The production of the different cytokines was determined by a commercially available ELISpot assay. In brief, ELLIP plates (Millipore, cat. no MAIPSWU) with PVDF membranes were treated with 70% ethanol for 1 minute, washed in sterile water and coated o/n at +4°C with 10 µg/mL of monoclonal antibodies specific for IFN-γ (AN18), IL-2 (1A12), IL-4 (11B11) or IL-5 (TRFK5) (Mabtech AB, Nacka strand, Sweden) in PBS. After washing 5 times in PBS, the plates were blocked for 2 hours with complete RPMI-1640 medium. All stimulations (36 hours at 37°C, 5% CO_2_) were carried out using 250,000 immune cells/well. Various concentrations of the different antigens were added in triplicates to a total volume of 200 µL. After stimulation, the wells were washed and incubated for 2 hours at 37°C with the following biotinylated antibodies, respectively: anti-IFN-γ (R4-6A2-biotin), anti-IL-2 (5H4-biotin), anti-IL-4 (BVD6-24G2-biotin) and anti-IL-5 (TRFK-biotin) (Mabtech AB) at 2 µg/mL in 0.5% FBS/PBS (Sigma Aldrich, F6178). After washing, Strep-ALP (Mabtech AB, 3310-10) diluted 1∶1,000 in 0.5% FBS/PBS was added and incubated for 1 hour in RT. Sterile-filtered substrate, BCIP/NBT (Mabtech AB), was used to develop spots; IFN-γ and IL-2 for 10 minutes, IL-4 for 12 minutes and IL-5 for 14 minutes. The substrate reaction was stopped by rinsing extensively with dH_2_O, after which the plates were left to dry. The number of spots was counted using the AID ELISpot reader and software ver. 3.2.3 (AID, Strassberg, Germany). The number of spots (cytokine producing cells) was determined at each concentration of peptide or protein and the results given as the number of IFN-γ, IL-2, IL-4 or IL-5-producing cells per 10^6^ cells. A mean number of cytokine-producing cells of <50 per 10^6^ cells was considered as negative.

### CTL and Th cell-proliferation in response to OVA and OVA peptides

Proliferative responses to OVA and OVA peptides were determined by stimulation of splenocytes from groups of mice immunized with the different vaccine compositions. A total of 600,000 cells/well in complete RPMI-1640 medium were seeded in 96-well flat bottom plates with lid (Corning, 3595). Stimulation was carried out for 48 hours at 37°C in a 5% CO_2_ atmosphere using the same antigens and concentrations as in the ELISpot assay. At 48 hours, 0.1 Ci/mL ^3^H-thymidine (TRA120-5MC; GE Heathcare, Chalfont St. Giles, United Kingdom) was added and 16–20 hours later the cells were harvested onto filtermat A filters (Wallac,145–421) and the radioactivity counted in a TRILUX 1450 MicroBeta counter (Wallac). Proliferation was determined by dividing the radioactivity as counts per minute (cpm) of cells incubated with an Ag with the cpm of the cells incubated with medium alone (sample to negative (S/N) ratio). Groups were compared by the mean S/N ratios at each time point after subtraction of proliferative responses seen in the negative control group receiving PPM alone. All samples were run in triplicates.

### Statistical analyses

The ANOVA with Tukey post-hoc test was used for statistical analyses using the JMP version 8.0.1 for PC software (SAS Institute Inc., Cary, North Carolina, USA). P-values <0.05 were considered statistically significant.

## Results

### Conjugation of OVA to mannosylated PSGL-1/mIgG_2b_ boosts anti-OVA IgG responses

**Figure 2:**
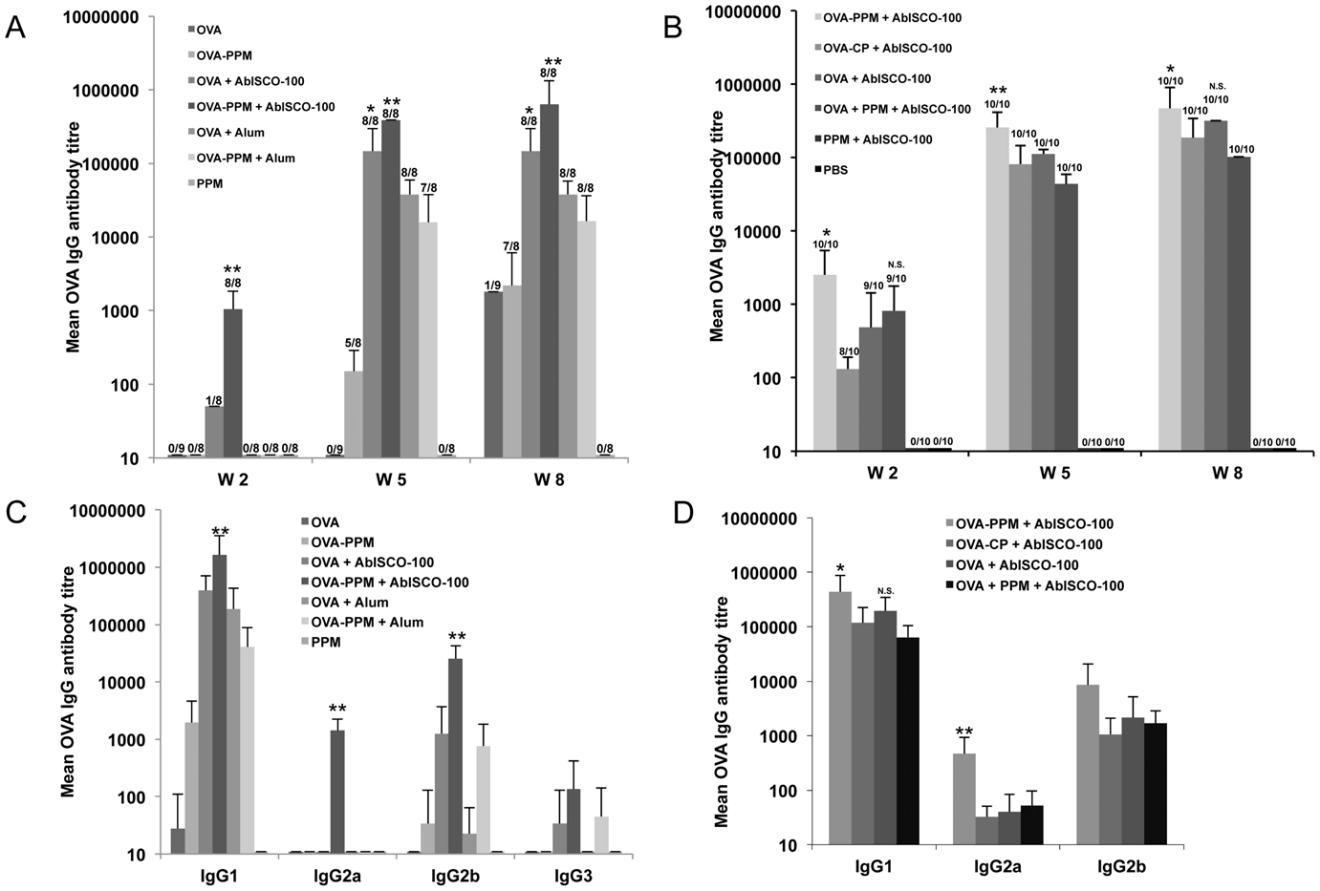
OVA specific IgG antibody titers. Serum IgG antibody titers against OVA in study A (**A**) and B (**B**). Titres are given as mean +SD (n = 8–9 in study A and 8–10 in study B) and are defined as the reciprocal endpoint dilution giving an optical density at 450 nm of 3 times (**A**) or ≥0.2 above (**B**) the background value. In study A (A), the asterisk (**) indicates a significantly higher IgG titer compared to all other groups and (*) indicates a significantly higher IgG titer compared to all groups except OVA−PPM+AbISCO-100 at that time point. In study B, the asterisk (*) indicates a significantly higher IgG titer compared to all other groups. P<0.05. Serum IgG-isotype antibody titers against OVA in study A (**C**) and B (**D**). Titres are given as mean +SD (n = 8–9 in study A and 8–10 in study B) and are defined as the reciprocal endpoint dilution giving an optical density at 450 nm of 3 times (**C**) or ≥0.2 above (**D**) the background value. The asterisk (**) indicates a significantly higher IgG titer compared to all other groups. P<0.05. An (*) indicates a significantly higher IgG titer compared to all groups except the group indicated N.S. (non significant) at that time point. P<0.05.

Total IgG was compared between all experimental groups at week 0 (data not shown), 2, 5 and 8 using mouse serum from individual mice. In study A (Fig. 2A) only the groups including the AbISCO®-100 adjuvant had detectable antibody titers at week two. The group that received OVA−PPM+AbISCO®-100 had significantly higher IgG titers as compared to all other groups. At week five after two immunizations, all groups except the group immunized with OVA alone had detectable IgG titers. Five out of eight mice immunized with OVA−PPM had IgG titers over 1∶100, while none of the mice immunized with OVA alone had detectable IgG titers. The OVA−PPM+AbISCO®-100 group had significantly higher titers compared to the OVA+AbISCO®-100 group. Imject®Alum showed a significantly weaker adjuvant effect on the induction of anti-OVA IgGs than AbISCO®-100. No difference in the anti-OVA IgG response could be detected between the two groups immunized with OVA+Imject®Alum or OVA−PPM+Imject®Alum at week five indicating that our mannosylated fusion protein does not potentiate the adjuvant effect of Imject®Alum. At week eight, after three immunizations, titers over 1∶1,000 was achieved in the group immunized with OVA−PPM. At this time point, the IgG pattern between the groups was similar to that seen at week five but with even higher IgG titers peaking 1∶10^6^ in the group immunized with OVA−PPM+AbISCO®-100.

In order to examine the importance of the oligomannose residues on the fusion protein for its adjuvant effect on the IgG antibody response, we included in study B a control fusion protein (CP) lacking oligomannose residues. At week two the group immunized with OVA−PPM+AbISCO®-100 had significantly higher antibody titers than the other groups including the group immunized with a fusion protein (CP) conjugate lacking oligomannose residues (Fig. 2B). However OVA−PPM+AbISCO®-100 was not significantly different from the OVA+PPM+AbISCO®-100 group. At week five the group that had received OVA conjugated to PPM+AbISCO®-100 had significantly higher IgG titers compared to all other groups. The adjuvant effect of PPM was also seen when it was mixed with OVA and AbISCO®-100 (Fig. 2B). At week eight the OVA−PPM+AbISCO®-100 group had significantly higher titers compared to all groups except the OVA+AbISCO®-100 group.

### The OVA-PSGL-1/mIgG2b conjugate induces a strong and broad anti-OVA IgG isotype response

The IgG isotype distribution within each group was evaluated at week eight using sera from individual mice (Fig. 2C and D). In study A, the IgG subclasses in the control group (PPM alone) were not analyzed since there were no anti-OVA IgG detected in this group. IgG1 followed by IgG2b was shown to be the most predominant IgG subclasses in all tested groups. IgG2a was found only in the group immunized with OVA−PPM+AbISCO®-100 with titres of 1∶1,000. Interestingly the other AbISCO®-100 group without the fusion protein had no detectable IgG2a at all. Anti-OVA IgG2b was detected with titers over 1∶10,000 in the OVA−PPM+AbISCO®-100 group that represents about ten times higher titers than in the OVA+AbISCO®-100 group. The same relationship was seen in the Imject®Alum groups where mice immunized with the fusion protein conjugate had titers of about 1∶1,000, while the OVA+Imject®Alum group had anti-OVA IgG2b titers below 1∶100. These results suggest that the mannosylated fusion protein supports an anti-OVA IgG2b response. Anti-OVA IgG3 titers were low or undetectable in all examined groups, peaking at a 1∶100 titer in the OVA−PPM+AbISCO®-100 group. The group immunized with OVA−PPM+AbISCO®-100 had significantly higher IgG1-, IgG2a- and IgG2b titres than all other groups.

In study B (Fig. 2D), the IgG subclasses in the control groups were not analyzed as no anti-OVA IgG were detected in these groups. Supportive of study A, IgG1 followed by IgG2b was shown to be the most predominant IgG subclasses in all tested groups. The OVA−PPM+AbISCO®-100 group had significantly higher IgG1 titers compared to all groups except the OVA+AbISCO®-100 group. IgG2a was again only found in the OVA−PPM+AbISCO®-100 group with titers of 1∶500 and this result was significantly higher than in the other groups. IgG2b was detected with titers of 1∶8,500 in the OVA−PPM+AbISCO®-100 group, which represents about five times higher titers than in the OVA+PPM+AbISCO®-100 group or the OVA+AbISCO®-100 group and eight times higher titers than the OVA−CP+AbISCO®-100 group. Anti-OVA IgG3 titers were low or undetectable in all examined groups (data not shown). These results suggest that the mannose structures in the fusion protein play a decisive role for an early onset of antibody production, for inducing high antibody titers and also for inducing an anti-OVA IgG2a response. When comparing conjugated OVA with just mixing, conjugation of OVA to PPM appears to give both stronger and broader antibody responses than when OVA is just mixed in with PPM.

### The PSGL-1/mIgG_2b_ fusion protein induces anti-mIgG responses

**Figure 3:**
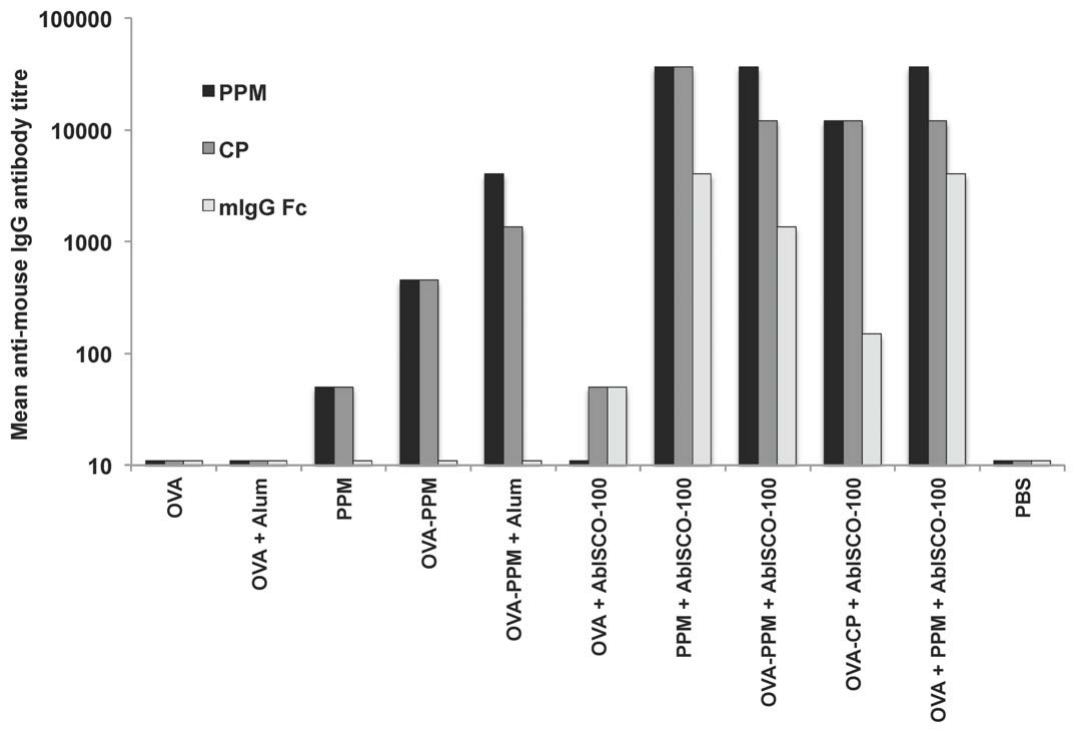
PSGL-1/mIgG_2b_ specific antibody titers. Serum IgG antibody titers against PPM, CP or mIgG Fc in groups of mice from study A and B. Titres were determined from pooled sera of mice in each group (8–10 mice/group) at week 8, and were defined as the reciprocal endpoint dilution giving an optical density at 450 nm of 3 times the background value.

Anti-mIgG responses were compared between the experimental groups at week 8 using pooled mouse sera from groups of mice included in study A and B (Fig. 3). The results show that all groups of mice immunized with the fusion protein (PPM or CP) have a detectable antibody response to *PSGL-1/mIgG_2b_*. Only groups receiving AbISCO®-100 developed antibodies to mIgG Fc fragments. In the groups immunized with PPM or CP together with AbISCO®-100, an enhanced IgG response to the fusion protein was detected. In general, higher IgG titers (>1∶10,000) were detected against PPM and CP as compared to the native mIgG Fc fragment for which the titers reached 1∶4,000.

### Induction of OVA-specific cytotoxic T-lymphocytes

**Figure 4:**
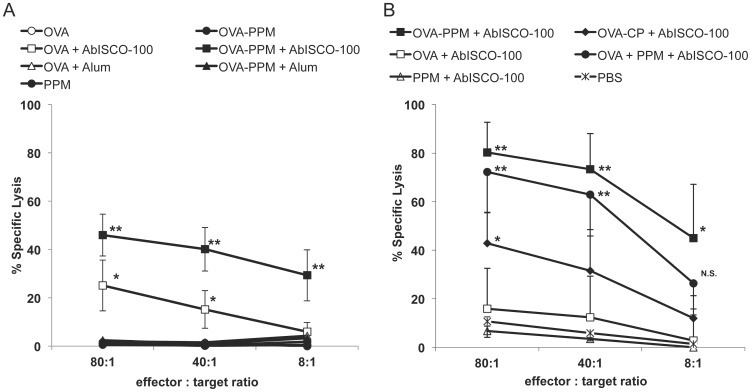
OVA-specific CTL responses. Detection of OVA peptide-specific cytolytic activity of splenocytes isolated from the different groups of mice and restimulated *in vitro* with an MHC class I-binding OVA peptide (SIINFEKL) in study A (**A**) and B (**B**). The results are presented as mean percentage specific lysis (n = 8–9 in study A and n = 4–5 in study B) of peptide coated RMA-S cells. The asterisks (**, *) indicates a statistical difference compared to the other groups at the specific effector∶target ratio. P<0.05. At effector∶target ratio 8∶1 in study B an (*) indicates a significantly higher specific lysis compared to all groups except the group indicated N.S. (non significant). P<0.05.

To study if the different immunizations were able to induce cellular immune responses with detectable OVA-specificity, lytic activity mediated by cytotoxic T lymphocytes were tested in ^51^Cr release assays using *in vitro* primed splenocytes. In this assay specific lysis of ^51^Cr-fed TAP-deficient cells (RMAs) loaded with an MHC class I-binding OVA peptide (SIINFEKL) and induced by peptide-stimulated splenocytes represent the effectiveness of an induced CTL response. The CTL activity was studied in individual mice within all groups (Fig. 4A and B). In study A, a specific lysis of target cells over 10%, was only detectable in groups of mice injected with compositions including the AbISCO®-100 adjuvant (Fig. 4A). Interestingly, there was significantly higher specific lysis in the OVA−PPM+AbISCO®-100 group compared to the group that received OVA+AbISCO®-100. At an 80∶1 effector∶target (E∶T) ratio, 25.1±10.5 lysis was detected in mice immunized with OVA+AbISCO®-100 as compared to 46.0±8.7; p<0.05 lysis in the group receiving OVA linked to the mannosylated fusion protein. At an 8∶1 E∶T ratio, the difference was even more clear with a specific lysis of 5.9±3.9 compared to 29.3±10.6; p<0.05.

In Study B, there was significantly higher specific lysis at E∶T ratios of 80∶1 and 40∶1 in the groups immunized with OVA−PPM+AbISCO®-100 (mean 80.2±12.5; p<0.05 lysis and 73.3±14.8; p<0.05 lysis, respectively) and OVA mixed with PPM+AbISCO®-100 (mean 72.2±16.6; p<0.05 lysis and 62.9±17.0; p<0.05 lysis, respectively) compared to the other groups (Fig. 4B). Although significantly lower than the OVA−PPM+AbISCO®-100 and OVA mixed with PPM+AbISCO®-100 groups, the OVA−CP+AbISCO®-100 group had a significantly higher CTL response (mean 42.8±12.6; p<0.05 lysis) at the 80∶1 E∶T ratio than the other groups. At the 8∶1 E∶T ratio, the group that received OVA−PPM+AbISCO®-100 had a mean specific lysis of 45±22.1; p<0.05, which was significantly higher compared to all groups except the OVA+PPM+AbISCO®-100 group (mean 26.4±10.6). These results support the antibody results and again show that the mannose structures in the fusion protein play a decisive role for inducing a broad immune response including a strong CTL response.

### Induction of OVA-specific T-cells producing IFN-γ, IL-2, IL-4 and IL-5

To further characterize and quantify the type of immune response elicited, we studied the *in vitro* secretion of IFN-γ, IL-2, IL-4 and IL-5. Spleen cells from a pool of splenocytes isolated from four to five immunized mice were stimulated for 36 hours with various antigens and the number of cells producing a particular cytokine was assessed by the ELISpot assay.

**Figure 5:**
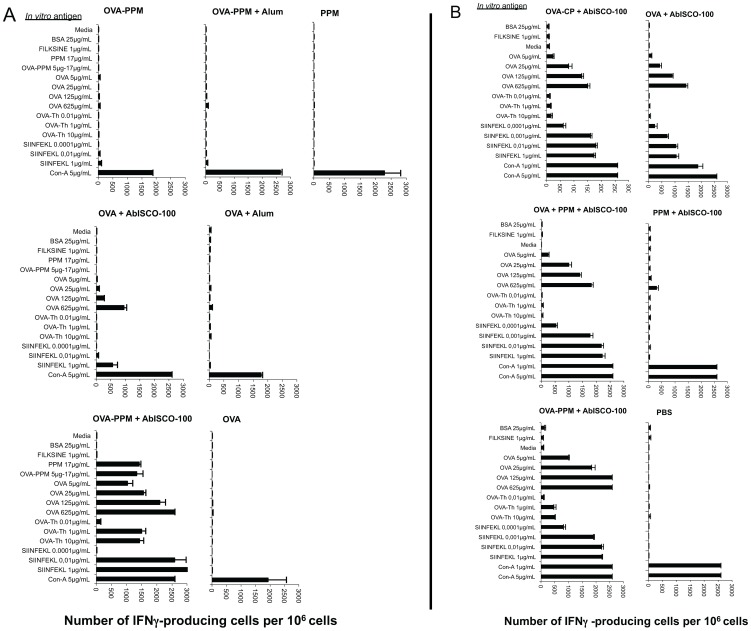
γ-IFN producing cells determined by ELISpot assay. Quantification of OVA-specific, γ-IFN-producing spot forming cells (SFC) after a 36 hour *in vitro* stimulation of splenocytes (pooled from 4–5 spleens) with indicated antigens [study A (**A**) and B (**B**)]. Data has been given as SFC/10^6^ splenocytes. More SFC than the cut-off of 50 SFC/10^6^ cells indicates a positive response.

In study A, IFN-γ producing cells were mainly seen in groups of mice immunized with compositions containing AbISCO®-100 (Fig. 5A). In the OVA+AbISCO®-100 group, IFN-γ producing cells were detected after stimulation with the MHC class I restricted SIINFEKL peptide (1 µg/mL) with up to 500 spot-forming colonies (SFC)/10^6^ cells. The corresponding value in the group of mice receiving the OVA−PPM+AbISCO®-100 was over 2,500 SFC/10^6^cells even when using 100 times lower concentration of the peptide (0.01 µg/mL). In the OVA−PPM+AbISCO®-100 group, not only IFN-γ producing CTLs but also Th-cells responded and secreted IFN-γ when stimulated with an OVA-Th peptide or the intact OVA protein. Although it seems that AbISCO®-100 is needed for the mice to mount a cellular immune response including induction of IFN-γ producing cells, the response is clearly higher when OVA is combined with the mannosylated PSGL-1/mIgG_2b_ than when it is used alone. Negative controls (FILKSINE, BSA and medium alone) did not induce any production of IFN-γ and the positive control (Con-A) showed similar responses between groups.

In study B, the IFN-γ response was shown to be highest in the group of mice immunized with OVA−PPM+AbISCO®-100, which was the only group that responded to the OVA-Th peptide (Fig. 5B). The response to the SIINFEKL peptide was similar in both groups that received OVA and PPM (conjugated or mixed)+AbISCO®-100. The group that received OVA conjugated to CP+AbISCO®-100 had about 500 spots less at 1 µg/mL SIINFEKL than these two groups, while the OVA+AbISCO®-100 group had about 1,000 spots less. The OVA−CP+AbISCO®-100 group showed a similar response to the group that received OVA+AbISCO®-100 alone, except in case of the SIINFEKL peptide. Negative controls (FILKSINE, BSA and medium alone) did not induce any production of IFN-γ and the positive control (Con-A) showed similar responses between groups.

**Figure 6:**
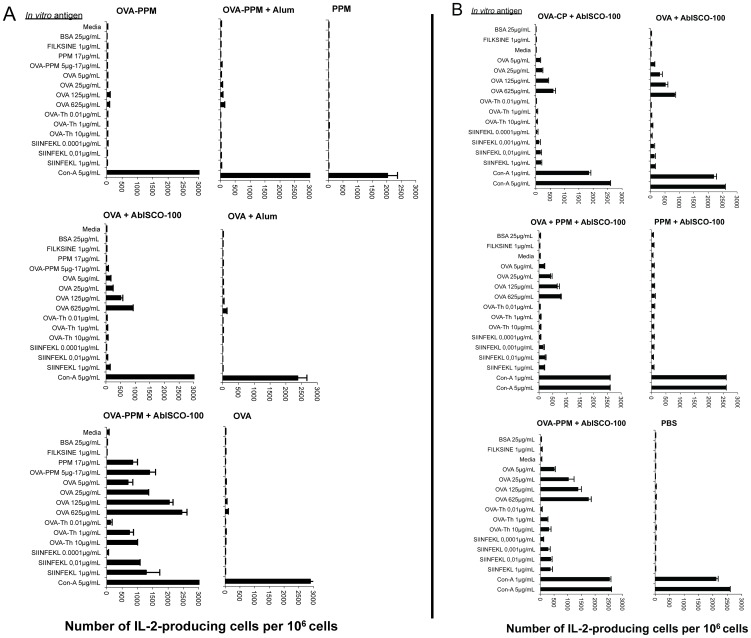
IL-2 producing cells determined by ELISpot assay. Quantification of OVA-specific, IL-2-producing spot forming cells (SFC) after a 36 hour *in vitro* stimulation of splenocytes (pooled from 4–5 spleens) with indicated antigens [study A (**A**) and B (**B**)]. Data has been given as SFC/10^6^ splenocytes. More SFC than the cut-off of 50 SFC/10^6^ cells indicates a positive response.

In study A, the differences observed with regard to the number of IFN-γ secreting cells were also seen when the number of IL-2 producing cells was assessed (Fig. 6A). Both stimulation with the MHC class II-binding OVA peptide and the MHC class I-binding peptide at a concentration of 1 µg/mL induced over 1,000 SFC when splenocytes from mice immunized with the mannosylated PSGL-1/mIgG_2b_ conjugate were used. In the OVA+AbISCO®-100 group, few (under 100 SFC) or no IL-2 producing SFC were detected. All controls behaved as expected.

In study B, the differences observed between the groups with regard to the number of IFN-γ secreting cells was not as clear when the number of IL-2 producing cells was assessed (Fig. 6B). The group that received the OVA conjugated to PPM+AbISCO®-100 again showed the highest response overall and was the only group that responded to the T-helper peptide. However, the other AbISCO®-100 and OVA containing groups showed similar responses when stimulated with OVA protein, MHC-I- (SIINFEKL) or MHC-II- (Th-peptide) binding peptide. All controls behaved as expected.

In relation to the Con-A control, the IL-4 ELISpot assay revealed fewer SFC as compared to both the IFN-γ and IL-2 ELISpot (Fig. S1A and S1B). There was also a slightly higher background with up to 50 SFC in the medium controls. However, there was a slight increase in the number of splenocytes secreting IL-4 in the OVA−PPM+AbISCO®-100 group following stimulation with recombinant OVA and the OVA Th peptide (1–10 µg/mL) suggesting an influence of Th-2 activation.

IL-5 producing cells were detected in low numbers and only when using OVA concentrations of 25–625 µg/mL (Fig. S2A and S2B).

### Proliferation

**Figure 7:**
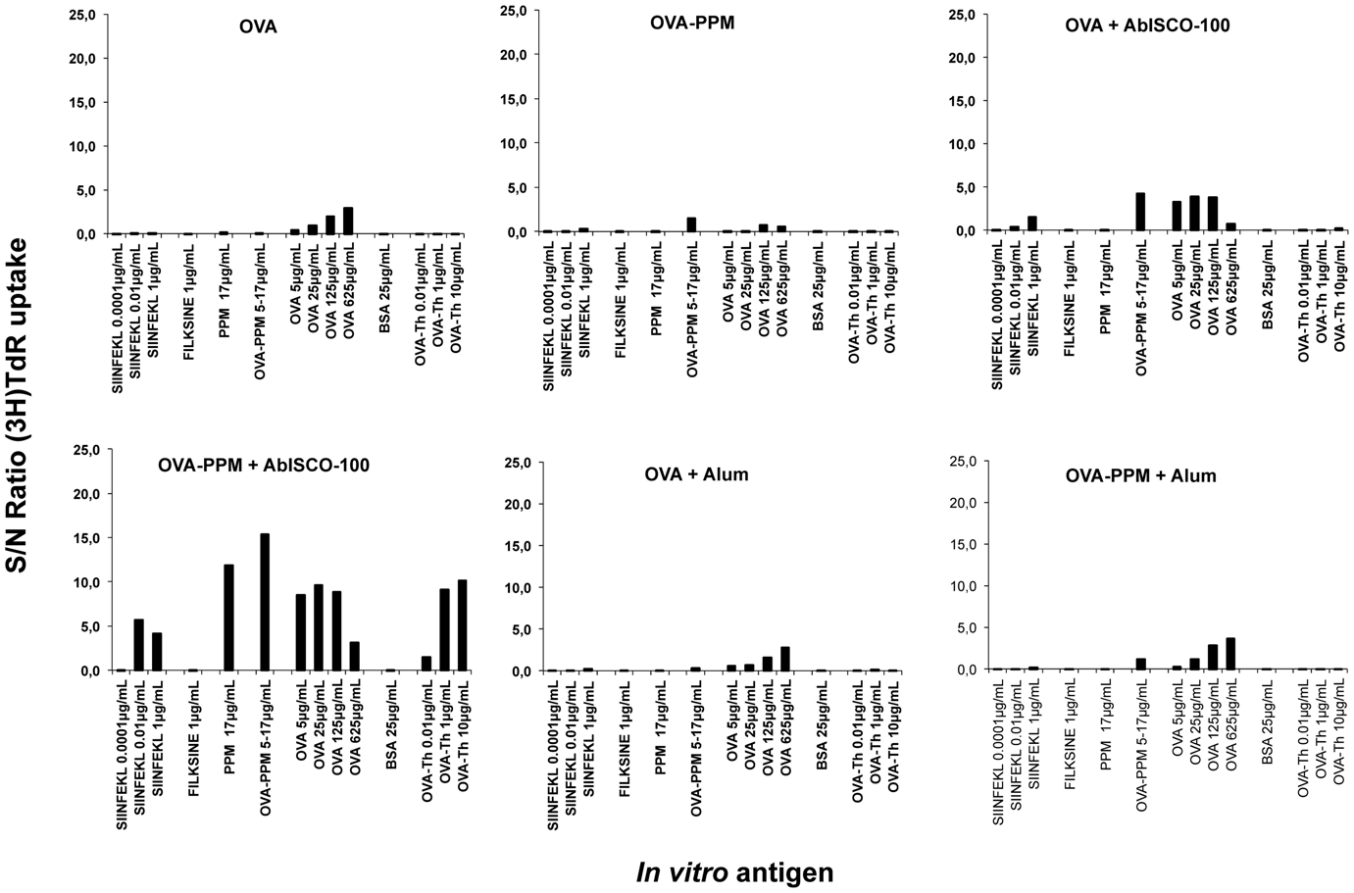
Proliferative OVA responses. Proliferative OVA-specific responses after a 68 hour stimulation of splenocytes (pools) with indicated antigens. The values in each group, presented as the S/N (sample to negative) ratio, indicates the difference between antigen-induced and spontaneous proliferation.

In an attempt to compare the proliferative responses between the groups, a ^3^H-thymidine proliferation assay was performed. In this assay, the ratio between the cpm value of splenocytes stimulated with the different antigens (in triplicate) and the cpm value of splenocytes incubated in medium alone was first calculated. These values were then compared to the proliferation seen in the control group immunized with PPM alone. Each graph describes the proliferative responses from a pool of splenocytes isolated from four mice within each group. The experiment was repeated twice with consistent results. The analysis revealed that OVA-specific proliferation was detectable mainly in the groups of mice immunized with antigen compositions containing AbISCO®-100 (Fig. 7). The OVA+AbISCO®-100 group mainly respond to stimulation with OVA protein whereas the OVA−PPM+AbISCO®-100 showed proliferative responses to OVA protein, OVA-Th peptide and OVA-CTL peptide stimulation. Consistent with the detection of anti-OVA antibodies, lytic CTLs and cytokine-producing (IFN-γ, IL-2) T-cells, the proliferative responses were higher when the OVA−PPM conjugate was used (Fig. 7). Alum did not have any effect on the CD4+ T-cell proliferation.

## Discussion

Numerous strategies have been suggested aiming at developing vaccine compositions targeting antigen presenting cells (APC) in order to improve antigen immunogenicity and elicit a Th1 response with the development of cytotoxic T lymphocytes (CTLs). The ability to generate CTL responses, and the killing of tumor cells and cells harboring intracellular pathogens, is maybe the most important feature of a therapeutic vaccine. One APC-directed strategy involves targeting mannose-binding receptors on macrophages and dendritic cells in order to improve vaccinogen uptake and MHC presentation (reviewed in [4], [32]). The engineered mucin-type immunoglobulin fusion protein, which when expressed in *Pichia pastoris* carried O-glycans comprised of linear oligomannose structures with up to nine residues and bound the mannose-specific receptors MMR, DC-SIGN and mannose-binding lectin (MBL) with high affinity [31], was evaluated here with regard to its effect on humoral and cellular anti-OVA immune responses *in vivo*.

In combination with AbISCO®-100, the OVA − mannosylated PSGL-1/mIgG_2b_ conjugate elicited a significantly faster and stronger antibody response compared to when OVA alone was used as antigen. Mannosylated PSGL-1/mIgG_2b_ improved the anti-OVA IgG response also without AbISCO®-100, but the response was weaker. The anti-OVA response was broader with regard to the IgG subclasses being induced and only the OVA − mannosylated PSGL-1/mIgG_2b_ conjugate with AbISCO®-100 induced an IgG2a response. IgG1 was the predominant IgG subclass detected suggesting an immune response skewed also towards a Th2 type of response. IgG2a and IgG2b antibody titers were, however, only detectable after inclusion of AbISCO®-100 and was stronger in the OVA − mannosylated PSGL-1/mIgG_2b_+AbISCO®-100 groups. These IgG subclasses would indicate a Th1 immune profile. The Th1 response is further evidenced by the generation of a strong OVA-specific CTL response and increased numbers of IFN-γ and IL-2 producing splenocytes in groups immunized with the OVA − fusion protein conjugate in the presence of AbISCO®-100. Increased CD4+ T-cell proliferation was also found, further confirming the ability of the OVA − mannosylated PSGL-1/mIgG_2b_+AbISCO®-100 combination to strongly activate the immune system. However the strong CTL response observed with the combination of OVA−PPM+AbISCO-100 was not seen when OVA−PPM was combined with Alum. Alum is the foremost vaccine adjuvant for clinical applications but it is a poor inducer of cellular immunity and the adjuvant mechanism of alum is not fully understood. Nevertheless in our study the combination with OVA−PPM+alum resulted in a somewhat broader humoral immune response compared to the OVA+alum group. It is thus evident that the combination of PPM and alum does not result in the strong synergy effect seen when PPM is combined with AbISCO-100. Further studies with PPM in combination with other adjuvant systems and/or antigens are necessary to evaluate PPMs adjuvant potency.

We found that groups of mice injected with the mucin-type fusion protein developed antibodies against the fusion protein irrespective of O-glycan substitution. Whether these antibodies were directed against the recombinant PSGL-1 part or the mouse IgG_2b_ Fc part is at the moment not clear. The fact that antibodies binding to the fusion protein itself were induced could limit its clinical use, especially upon re-utilization. However, such antibodies against the fusion protein may not necessarily have limitations. In fact, antibodies against the fusion protein may act to further improve antigen uptake and presentation. More serious, however, was the observation that groups receiving AbISCO®-100 developed antibodies reactive with mouse IgG Fc fragments, which indicates that self tolerance was broken. Low titers of antibodies against the CP55.2 fusion protein and mouse IgG was observed in the OVA alone+AbISCO®-100 group, suggesting that a weak polyclonal B cell activation might have occurred. Clinical relevance of such responses will need to be assessed in the clinical development of any AbISCO®-100 mannosylated PSGL-1 mIg-based candidate. Whether this can lead to autoimmunity remains to be seen.

The fact that mannosylated PSGL-1/mIgG_2b_ binds to MMR, DC-SIGN and MBL *in vitro* [31], and that the OVA − fusion protein conjugate triggered a faster and stronger anti-OVA IgG response than OVA alone (Fig. 2), suggests that the mannosylated mucin-type fusion protein also without AbISCO®-100 has an immune-stimulating effect. It is clear from the data though that addition of AbISCO®-100 is required in order to generate an anti-OVA CTL response (Fig. 4). But also in the presence of AbISCO®-100, the OVA − fusion protein conjugate triggered a stronger anti-OVA CTL response than OVA alone (Fig. 4). The AbISCO®-100 adjuvant is an immunostimulatory complex matrix (ISCOMATRIX™) consisting of a selection of purified fractions of Quillaja saponins formulated with a mixture of cholesterol and phosphatidyl choline. This adjuvant is known to induce a broad immune response with strong antigen-specific cellular and humoral immune responses, and have been tested with numerous antigens both in humans and veterinary vaccine designs [33], [34], [35]. The adjuvant mechanisms of ISCOMATRIX™ include the capability of antigen presentation by both MHC class I and MHC class II pathways [36] and the production of pro-inflammatory cytokines such as IL-12, IL-1, IL-6, IL-8 and IFN-γ with subsequent recruitment of lymphocytes [37], [38]. A recent study demonstrates that when OVA was incorporated in ISCOMATRIX forming ISCOMs this lead to a potent immune activation and antigen delivery to CD8α^+^ DCs *in vivo* with effective cross-priming of CD8^+^ T cells and subsequent efficient induction of CTL responses [39]. In our study the synergistic effect between the mannosylated fusion protein and AbISCO®-100 was also reflected in the higher frequency of IFN-γ- and IL-2-producing splenocytes recovered from mice immunized with the conjugate plus AbISCO®-100 compared to OVA alone plus AbISCO®-100 (Fig. 5 and 6, respectively). The results obtained suggest that the mannosylated fusion protein improve the immunogenicity of the conjugated OVA, but that addition of AbISCO-100® had a synergistic effect. Our hypothesis is that AbISCO-100® induces a local inflammatory reaction that stimulates recruitment and activation of antigen-presenting cells at the site of injection, and that conjugating OVA to the mannosylated mucin-type fusion protein improves antigen uptake by APC. Other studies with mannosylated liposomes have shown that there is a need for the addition of either Quil A, Alum or TLR-ligands as adjuvant in order to induce a potent activation of immune cells and upregulation of costimulatory receptors such as CD80 and CD86 [40], [41]. However, mannosylated dendrimers have been shown to induce maturation of bone marrow DCs and to upregulate CD80, CD86 and CD40 [42]. They further demonstrated in a B16-OVA tumor model that tumors in mice pre-immunized with mannosylated OVA dendrimers did not grow, or displayed a more delayed onset and had slower kinetics of growth, than those of OVA-immunized mice [42]. The same group also published a report suggesting that there is, in fact, a concomitant need for TLR signaling for optimal function of DC subsets in antigen localization, processing and presentation [43].

Mannose receptor-mediated uptake of antigen has been shown to improve T-cell presentation a 100-fold compared to fluid phase uptake [44]. Similarly, antigen uptake by the endocytic receptor DC-SIGN has been shown to direct antigen to the late endosomal/lysosomal compartments and improve CD4^+^ T-cell presentation [45]. Although mannose-specific endocytic receptors may facilitate the transport of OVA to the compartments where antigen processing and MHC loading can occur, other processes may be involved which governs MHC loading. For example, it has been shown that the efficiency of antigen presentation on MHC class II molecules is dependent on the co-occurrence of Toll-like receptor (TLR) ligands and antigen in the same phagosome [46]. Furthermore, it has been argued that TLR signaling might influence phagosome maturation in such a way as to remodel the late endosomal/lysosomal compartments for efficient antigen processing and MHC II loading [47]. The question remains whether the O-glycan oligomannoses of the fusion protein are able to directly engage TLR:s. There are reports on TLR4 recognizing mannans from *Saccharomyces cerevisiae* and *Candida albicans* [48], and that short linear O-linked mannans of *C. albicans* are recognized by TLR4 and induce proinflammatory cytokine production, such as TNF-α [49]. Though a recent study showed that only some *C. albicans* strains were recognized by TLR4 [50].

A role for mannose-binding receptor targeting and enhanced antigen uptake is also suggested by the fact that O-glycan oligomannoses are required on PSGL-1/mIgG_2b_ for an optimal immune-stimulating effect. When OVA was conjugated to a fusion protein expressed in CHO cells and carrying mono and disialylated core 1 structures, weaker humoral and cellular anti-OVA responses were detected. When comparing conjugated OVA with just mixing, conjugation of OVA to mannosylated PSGL-1/mIgG_2b_ appear to give more rapid, stronger and broader antibody responses than when OVA is just mixed with mannosylated PSGL-1/mIgG_2b_.

Antigen-specific CTL activities are important for control of virus infected cells and tumors [51], [52], [53]. Recombinant antigens frequently do not elicit CTL responses, possibly due to low incidence of MHC I presentation for exogenously internalized antigens [54]. However, under certain conditions and with some antigens cross-presentation may be more pronounced, which could serve to improve CD8+ T cell activation [55]. When conjugated to OVA and if given together with AbISCO®-100, the mannosylated fusion protein appears to be able to skew the anti-OVA response towards a Th1 response and the generation of OVA-specific CTL:s. In addition, IgG2a antibody titers were only detectable in the group that received the OVA − mannosylated PSGL-1/mIgG_2b_ conjugate together with AbISCO®-100. This suggests that OVA peptides may be more efficiently cross-presented when the OVA − mannosylated fusion protein conjugate is processed in APC. Alternatively, the conjugate stimulates cytokine (IL-12) secretion from APC that potentiates differentiation of activated Th cells to Th1 cells. Oxidized mannan coupled to MUC1 has been found to activate macrophages leading to IL-12 secretion [13]. Other *in vitro* studies have found that OVA, when carrying multiple O-glycosylation sites and expressed in *P. pastoris*, is more potent in inducing CD8+ T-cell proliferation than when *P. pastoris*-expressed OVA carries mixed N- and O-glycans or N-glycans alone [16]. The majority of PSGL-1/mIgG_2b_ glycans are O-glycans. Hence, extensive O-mannosylation may be particularly important for eliciting Th1 type of responses. Because the O-glycans of the mannosylated OVA used in the previous study were not characterized, it is difficult to try to identify an O-glycan determinant responsible for this effect. Collectively, the characterizations of O-glycans derived from *P. pastoris*-produced glycoproteins performed so far have demonstrated diversity and to suggest that *P. pastoris*-derived O-glycans have similar structures on different proteins may be misleading. *P. pastoris* O-glycans include Hex_2–9_ structures, with or without phosphorylation, α1,2 and/or α1,3 glycosidic linkages, as well as terminal or subterminal mannoses linked by β1,2 glycosidic linkages [31], [56], [57]. We have shown with surface plasmon resonance techniques that PSGL-1/mIgG_2b_ binds with similar high binding affinities to recombinant MBL, MR and DC-SIGN [31]. These results indicate that all of these receptors might be targeted *in vivo*. However, the specific signaling from one receptor and its contribution to subsequent events leading to the final immunological outcome of ligand binding is hard to assess. In one study MR^−/−^ mice were used to demonstrate that the mannose receptor could direct soluble OVA for cross-presentation by dendritic cells suggesting that MR may have contributed to the enhanced CTL-activities observed in this study [20]. This is also supported by other studies, which have suggested that targeting the MR by MUC1 coupled to oxidized mannan was important for obtaining high frequency anti-MUC1 CTL responses [58]. On the other hand, cross-talk with TLR:s by for example MBL and/or DC-SIGN may also gear the adaptive immune response towards a Th1 reaction making it difficult to assign one particular receptor to the final immunological outcome.

In addition to the mentioned receptors, other lectins may also be involved. For example, Dectin-1 belongs to the C-type lectins like MBL, MR and DC-SIGN and has been shown to bind cell wall components and beta-glucans of fungal pathogens including *C. albicans* [59]. Dectin-1 can induce DC maturation, which subsequently may potentiate the differentiation of naïve CD4+ T cells to IL-17 secreting Th17cells important for anti-fungal responses [60], [61]. It is interesting to speculate that Dectin-1 may be involved in the shaping of the anti-OVA immune responses observed in the present study. However, it has been noted that Th1-associated cytokines repress Th17-differentiation in the mouse. Consequently, the anti-OVA Th1 type of responses elicited by the OVA−PPM conjugate in this study would contradict involvement of Dectin-1 [62], [63]. In addition, the O-glycans of *P. pastoris* derived PSGL-1/mIgG_2b_ are not identified as ligands for Dectin-1 [31], [59]. Assaying for IL-23 and/or IL-17 amongst the splenocytes and lymph node cells would perhaps reveal involvement of Th17 cells and the Dectin-1 receptor.

## Conclusions

In conclusion, we have shown that the mannose structures in the fusion protein play a decisive role for inducing a broad immune response with a rapid and strong antibody response and a strong CTL response. When comparing conjugated OVA with just mixing, conjugation of OVA to mannosylated PSGL-1/mIgG_2b_ appear to give a more rapid, stronger and broader antibody response than when OVA is mixed with mannosylated PSGL-1/mIgG_2b_. Hence this study demonstrates that PSGL-1/mIgG_2b_ produced in *P. pastoris* conjugated or just mixed with an antigen may indeed improve the antigen specific immune responses *in vivo*. This immune enhancing effect appear to be synergistic with AbISCO®-100, but not with Alum. Conjugation of the mannosylated fusion protein to an antigen may be particularly interesting when a Th1 type of response is desired as in the case of therapeutic vaccination against cancer. Further studies are needed to verify if sufficient B and T cell memory is induced, which is necessary for vaccination against viral infections.

## Supporting Information

Figure S1 IL-4 producing cells determined by ELISpot assay. Quantification of OVA-specific, IL-4-producing spot forming cells (SFC) after a 36 hour *in vitro* stimulation of splenocytes (pooled from 4–5 spleens) with indicated antigens [study A (**A**) and B (**B**)]. Data has been given as SFC/10^6^ splenocytes. More SFC than the cut-off of 50 SFC/10^6^ cells indicates a positive response.
(TIFF)

Figure S2 IL-5 producing cells determined by ELISpot assay. Quantification of OVA-specific, IL-5-producing spot forming cells (SFC) after a 36 hour *in vitro* stimulation of splenocytes (pooled from 4–5 spleens) with indicated antigens [study A (**A**) and B (**B**)]. Data has been given as SFC/10^6^ splenocytes. More SFC than the cut-off of 50 SFC/10^6^ cells indicates a positive response.
(TIFF)
